# The Proliferation of Pre-Pubertal Porcine Spermatogonia in Stirred Suspension Bioreactors Is Partially Mediated by the Wnt/β-Catenin Pathway

**DOI:** 10.3390/ijms222413549

**Published:** 2021-12-17

**Authors:** Sadman Sakib, Anna Voigt, Nathalia de Lima e Martins Lara, Lin Su, Mark Ungrin, Derrick Rancourt, Ina Dobrinski

**Affiliations:** 1 Department of Biochemistry and Molecular Biology, Cumming School of Medicine, University of Calgary, Calgary, AB T2N 4N1, Canada; sadman.sakib@ucalgary.ca (S.S.); rancourt@ucalgary.ca (D.R.); 2 Department of Comparative Biology and Experimental Medicine, Faculty of Veterinary Medicine, University of Calgary, Calgary, AB T2N 1N4, Canada; anna.voigt1@ucalgary.ca (A.V.); nathalia.delimaemart@ucalgary.ca (N.d.L.e.M.L.); lsu@ucalgary.ca (L.S.); mdungrin@ucalgary.ca (M.U.); 3 Biomedical Engineering Graduate Program, University of Calgary, Calgary, AB T2N 1N4, Canada; 4 Department of Oncology and Medical Genetics, Cumming School of Medicine, University of Calgary, Calgary, AB T2N 4N2, Canada

**Keywords:** bioreactor, stirred suspension bioreactor, germ cell, spermatogonia, culture

## Abstract

Male survivors of childhood cancer are at risk of suffering from infertility in adulthood because of gonadotoxic chemotherapies. For adult men, sperm collection and preservation are routine procedures prior to treatment; however, this is not an option for pre-pubertal children. From young boys, a small biopsy may be taken before chemotherapy, and spermatogonia may be propagated in vitro for future transplantation to restore fertility. A robust system that allows for scalable expansion of spermatogonia within a controlled environment is therefore required. Stirred suspension culture has been applied to different types of stem cells but has so far not been explored for spermatogonia. Here, we report that pre-pubertal porcine spermatogonia proliferate more in bioreactor suspension culture, compared with static culture. Interestingly, oxygen tension provides an avenue to modulate spermatogonia status, with culture under 10% oxygen retaining a more undifferentiated state and reducing proliferation in comparison with the conventional approach of culturing under ambient oxygen levels. Spermatogonia grown in bioreactors upregulate the Wnt/ β-catenin pathway, which, along with enhanced gas and nutrient exchange observed in bioreactor culture, may synergistically account for higher spermatogonia proliferation. Therefore, stirred suspension bioreactors provide novel platforms to culture spermatogonia in a scalable manner and with minimal handling.

## 1. Introduction

Advances in cancer therapies have led to increased survival rates of pediatric cancer patients [1,2]. As a result, there is a growing number of childhood cancer survivors who suffer from the late effects of gonadotoxic chemotherapy, which include hypogonadism and infertility due to loss of the spermatogonia pool [3,4]. Although cryopreservation of sperm is a routine fertility preservation technique for adult men undergoing chemotherapy, there is no such option for pre-pubertal boys before the onset of spermatogenesis. This has led to an interest in the isolation and cryopreservation of testicular biopsies, to allow subsequent isolation, expansion, and transplantation of spermatogonia back into the testes to restore spermatogenesis [5]. 

Although the culture of rodent spermatogonia is well established [6], scalable expansion of non-rodent spermatogonia in culture has had limited success [7,8,9,10]. Most of the research reported so far has employed conventional static culture systems, which are labor intensive and can be challenging to scale. Further, as “open” culture systems that involve extensive handling, they are particularly vulnerable to contamination and variability between batches, factors that can further reduce their utility in clinical applications [11,12]. Another important avenue of cell culture that is often neglected in the majority of reports is O_2_ tension [13,14]. In most cases, it is assumed that incubators set up for ambient O_2_ tension deliver 20.9% O_2_ to the cells. In reality, the O_2_ levels the cells experience depend on a number of factors such as altitude, media height, type of cell being cultured, and absence or presence of agitation [13,14]. Diffusive barriers in static culture further reduce O_2_ levels, often quite dramatically [13]. Previous research has shown that a reduced O_2_ tension environment is more supportive to several types of stem cells than culture under ambient O_2_ tension [15,16,17]. Recent studies also indicated that low O_2_ tension of 10% is more supportive for mouse and cattle spermatogonia proliferation in vitro [10,18].

We, therefore, assessed the potential for stirred suspension bioreactors (SSBs) to address the need for scalable expansion of spermatogonia within a controlled environment at ambient and low (10%) O_2_ tension. As the availability of immature human testis tissue for research is severely limited, we used spermatogonia from pre-pubertal pigs, which are widely available and share many physiological similarities with humans [19,20,21,22]. SSBs are sealed glass chambers with a central (generally magnetically driven) impeller, as well as ports for gas and medium exchange. Speed-controlled rotation of the impeller provides mixing and controls the shear forces to which the culture is exposed. The result is a scalable, controlled culture environment, with enhanced homogeneity, oxygen and mass transfer, and reduced variability [12]. We and others have demonstrated efficient and effective expansion of pluripotent stem cells in SSBs [23,24,25] and a role for upregulation of the Wnt/β-catenin pathway via the shear forces produced in SSB culture [25,26,27,28]. This is consistent with reports that fluid shear forces increase E-cadherin expression, induce adherens junctions [29], and upregulate the Wnt/β-catenin pathway and downstream C-myc expression [28]. Notably, Wnt/β-catenin signaling has also been shown to support the proliferation of murine spermatogonia [30].

Here, we report a novel suspension culture model for the scalable expansion of porcine pre-pubertal spermatogonia using the SSB. We found that while 10% O_2_ tension supports less proliferation than ambient O_2_ levels, it retains more undifferentiated spermatogonia. We also found that shear forces generated in the SSB upregulate the Wnt/β-catenin pathway, which is partially responsible for the increased proliferation of porcine spermatogonia in suspension culture. 

## 2. Results

### 2.1. Rotation Speed of 120 rpm (3.9 dyne/cm^2^) Supports the Highest Level of Spermatogonia Proliferation

**Figure 1:**
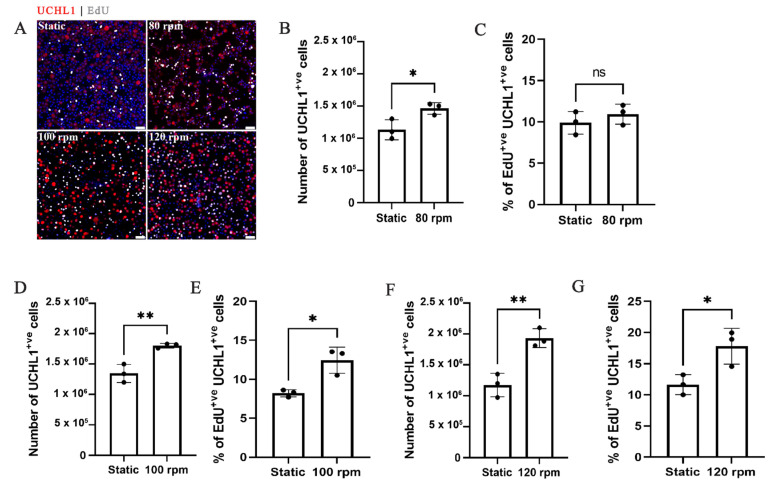
Stirred suspension bioreactor culture at 120 rpm (3.9 dyne/cm^2^) supports the highest level of spermatogonia proliferation: (**A**) immunofluorescence images of spermatogonia after static, 80, 100, and 120 rpm SSB culture. Scale bars 50 µm; (**B**,**D**,**F**) number of spermatogonia harvested after 2 weeks of SSB and static culture at 80 (**B**) 100 (**D**), and 120 (**F**) rpm; (**C**,**E**,**G**) percentage of proliferating spermatogonia after 2 weeks of SSB and static culture 80 (**C**), 100 (**E**), and 120 (**G**) rpm. *n* = 3, mean ± SD. *p* > 0.05 (ns), * *p* ≤ 0.05, ** *p* ≤ 0.01.

Cell populations from 1-week-old porcine testes were enriched for spermatogonia (82.2 ± 1.6%) positive for the spermatogonia marker ubiquitin *C*-terminal hydrolase L1 (UCHL1) [31,32] and cultured in 10 mL SSBs (Figure S1A) at 60, 80, 100, 120, and 140 rpm, alongside static-culture controls, for 14 days. These rotation speeds were selected based on previously reported studies [23,25,33,34,35,36] and translate to maximum shear forces of 1.5, 2.2, 3.0, 3.9, and 4.8 dyne/cm^2^, respectively (Figure S1B). The viability of the cells in static, 60, 80, 100, 120, and 140 rpm bioreactors were, respectively, 87.6 ± 2.6% (*n* = 15), 42.2 ± 7.7%, 90.4 ± 1.5%, 92.5 ± 2.3%, 93.7 ± 2.6% and 50 ± 5.6% (*n* = 3). As the viability of cells cultured at 60 and 140 rpm SSBs were low, these rotation speeds were excluded from future experiments and analyses. The number of spermatogonia and percentages of proliferative spermatogonia was analyzed by immunofluorescence for UCHL1 and EdU incorporation. Compared with the respective static cultures, SSB cultures (80, 100, and 120 rpm) had overall higher total spermatogonia yields (*n* = 3, *p* < 0.05) (Figure 1B,D,F). Similarly, the percentage of proliferative spermatogonia (EdU^+ve^ UCHL1^+ve^ cells) was higher in all SSB cultures except 80 rpm (*n* = 3, *p* < 0.05) (Figure 1A,C,E,G). Of the three SSB rotation speeds, 120 rpm that had a maximum shear force of 3.9 dyne/ cm^2^, supported the highest degree of proliferation (Figure S1C), and as a result, only 120 rpm rotation speed SSB cultures were used for further experiments.

Interestingly, mouse spermatogonia cultured under similar conditions had substantially reduced viability (static culture: 92.1–93.4%, 120 rpm: 8–10%, *n* = 2; 60 rpm: 10%, 80 rpm: 12.1%, *n* = 1) and proliferation (static culture: 15.2–18.2%, 120 rpm: 4.1–8.3%, *n* = 2; 60 rpm: 5.3%, 80 rpm: 4.8%, *n* = 1) in SSB. As a result, mouse spermatogonia cultures in SSBs were not pursued further. 

### 2.2. In Suspension Culture, Porcine Spermatogonia Proliferate More in Ambient O_2_ Tension but Contain Fewer Undifferentiated Spermatogonia

Mouse and cattle spermatogonia cultures at 10% O_2_ support better expansion in vitro [10,18]. It should be noted that incubators filled with ambient air are widely referred to as containing 20.9% O_2_; however, dilution with CO_2_ and water vapor results in actual oxygen content that is somewhat lower. At sea level, this reduction is to ~18.6% O_2_ in the gas phase; in our laboratory in Calgary, elevation (~1084 m) reduced air pressure and consequently oxygen levels as well (see Supplementary Methods for detailed calculations) [13,14,37,38]. To explore if low O_2_ tension would be beneficial for porcine spermatogonia culture, cells from 1-week-old pigs (containing 82.2 ± 1.6% UCHL1^+ve^ cells) and from 8-week-old pigs (containing 80.6 ± 1.4% UCHL1^+ve^ cells) were cultured in static plates (control) and in 120 rpm SSBs at 10% and ambient O_2_ tensions. The 10% O_2_ and the ambient culture conditions translate to 3.45 mM and 6.34 mM of O_2_ in the gas phase (Table 1). 

The O_2_ tension and concentration in the liquid phase (media) at 48 h as measured via Oxygen Sensor Spots SP-PSt3-NAU (PreSens Regensburg, Germany) (Figure S2C, Supplementary Methods) [32] are shown in Table 2.

**Figure 2:**
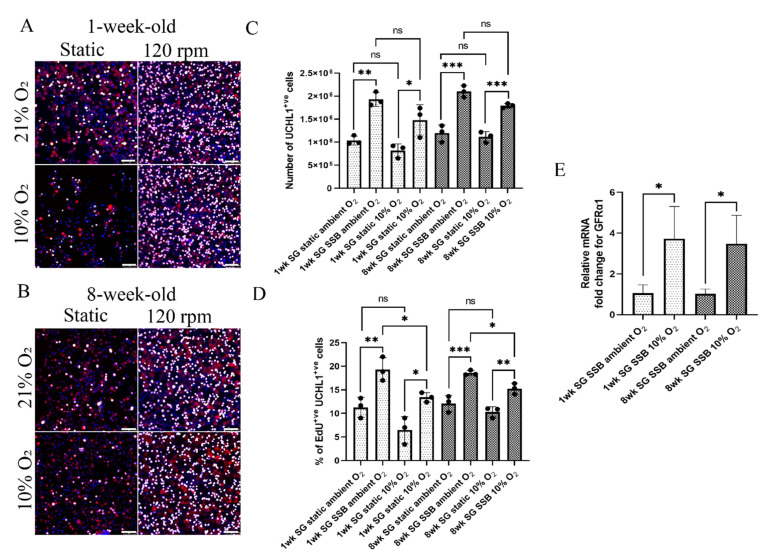
Spermatogonia cultured at 10% O_2_ tension proliferate less than those cultured at ambient O_2_ tension but contain more undifferentiated cells: (**A**,**B**) immunofluorescence images of 1-week-old and 8-week-old spermatogonia after 14 days of culture: red, UCHL1; grey, EdU; blue, DAPI. Scale bars 75 µm; (**C**) number of 1-week-old and 8-week-old spermatogonia (SG) harvested after 14 days of culture; (**D**) percentage of proliferating 1-week-old and 8-week-old spermatogonia (SG) after 14 days of culture; (**E**) relative mRNA fold change of *GFRα1* for 1-week-old and 8-week-old spermatogonia (SG). *n* = 3, mean ± SD. *p* > 0.05 (ns), *p* ≤ 0.05 (*), *p* ≤ 0.01 (**), *p* ≤ 0.001 (***).

Both 1- and 8-week-old spermatogonia were examined to evaluate the effect of SSB culture on early and late pre-pubertal spermatogonia, as we reported previously that these cells have different metabolic requirements [32]. For 1-week-old spermatogonia, total cell yield after 2 weeks of culture was higher in both 10% and ambient O_2_ (*n* = 3, *p* < 0.05) tension, compared with static culture (Figure 2C). However, no significant difference in cell yield was observed between SSB cultures in ambient and 10% O_2_ tension (Figure 2C). Similarly, proliferation levels (% of EdU^+ve^ UCHL^+ve^ cells) were higher for SSB cultures, compared with static controls for both O_2_ tensions (*n* = 3, *p* < 0.05). In addition, spermatogonia proliferation was higher in ambient O_2_ SSB culture, compared with 10% O_2_ culture (*n* = 3, *p* < 0.05) (Figure 2A,D). 

For 8-week-old spermatogonia, similar to 1-week-old cells, cell yield after 2 weeks of culture was higher in SSBs at both 10% and ambient O_2_ (*n* = 3, *p* < 0.05) tension, compared with the static. Cell yield between SSB cultures in 10% and ambient O_2_ tension was not significantly different (Figure 2C). Spermatogonia proliferation was higher for SSB cultures, compared with static controls at both 10% and ambient (*n* = 3, *p* < 0.05) O_2_ cultures (Figure 2B,D). Compared with 10% O_2_ tension, spermatogonia proliferation was higher in ambient O_2_ tension for SSB cultures (*n* = 3, *p* < 0.05) (Figure 2D). 

It has been reported that mouse spermatogonia culture at ambient O_2_ results in differentiation into progenitor cells and loss of stem cell potential [6,18,39]. To elucidate if the spermatogonia in SSB culture are undergoing differentiation, the transcription of GDNF family receptor alpha 1 (*GFRα1*) [40] and promyelocytic leukemia zinc finger (*PLZF*), as markers of undifferentiated spermatogonia, and deleted in azoospermia-like (*DAZL*), a marker of differentiating spermatogonia [41], was evaluated. Although no significant difference in transcription levels was observed for *PLZF* and *DAZL* (Figure S2A,B), *GFRα1* transcription levels were 3.72-fold higher in 1-week-old cells and 3.18-fold higher in 8-week-old cells (*n* = 3, *p* < 0.05) cultured at 10% O_2_ tension, compared with cells cultured under ambient O_2_ (Figure 2E) indicating retention of a more undifferentiated state, which is consistent with previous reports [6,18,39]. Subsequent analyses were therefore carried out at 10% O_2_ tension. 

### 2.3. Shear Forces Generated in SSB Culture Activate the Wnt/β-Catenin Pathway

**Figure 3:**
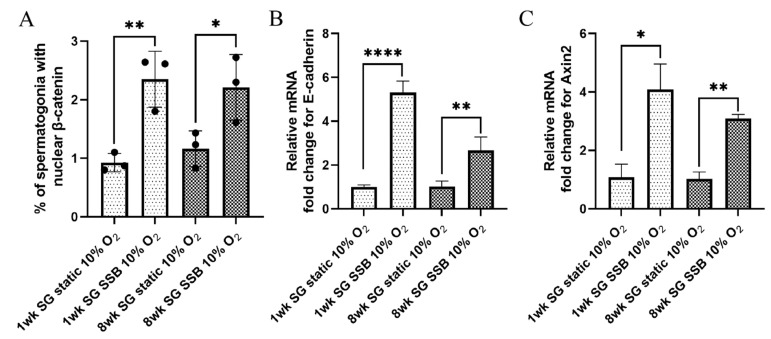
Suspension bioreactor culture elicits Wnt/β-catenin pathway activation: (**A**) percentage of 1- and 8-week-old spermatogonia (SG) with nuclear β-catenin; (**B**) relative mRNA fold change of *E-cadherin* for 1- and 8-week-old spermatogonia (SG); (**C**) relative mRNA fold change of *Axin2* for 1- and 8-week-old spermatogonia (SG). *n* = 3, mean ± SD. *p* > 0.05 (ns), *p* ≤ 0.05 (*), ** *p* ≤ 0.01, **** *p* ≤ 0.0001.

Fluid shear forces are known to activate the Wnt/β-catenin pathway by nuclear translocation of β-catenin [25,28,42,43,44], likely mediated by the mechano-sensing properties of E-cadherin [45]. β-catenin binds to the cytoplasmic tail of E-cadherin [46], which, upon exposure to fluid shear in SSBs, is dislodged from the adherens junction and accumulates in the cytoplasm resulting in nuclear translocation and pathway activation [25]. To investigate Wnt/β-catenin pathway activation, 1- and 8-week-old cells cultured in static conditions and SSBs at 120 rpm under 10% O_2_ tension were stained for β-catenin (1:200, Abcam) and vimentin, a marker for Sertoli and interstitial cells (1:500, Sigma) (Figure S3A,B), and the number of vimentin^-ve^ cells with nuclear β-catenin were counted to determine the % of spermatogonia displaying nuclear β-catenin localization. For both age groups, the percentages of spermatogonia with nuclear β-catenin were higher in SSB cultures, compared with static controls (*n* = 3, *p* < 0.05) (Figure 3A, Figure S3C–H). Spermatogonia express E-cadherin [47] and grow as clusters in the SSBs (Figure S3I,J). This clustering is mediated by E-cadherin [48], which likely serves as an upstream mediator for the nuclear localization of β-catenin in SSBs. To further characterize the Wnt/β-catenin pathway activation, qPCR for *E-cadherin* and *Axin2*, a downstream target of the Wnt/β-catenin pathway [49], was performed. Transcription data revealed that both *E-cadherin* and *Axin2* were upregulated in SSB culture, compared with conventional static culture, with *E-cadherin* increasing 5.3-fold for 1-week-old and 2.7-fold for 8-week-old cells, while *Axin2* was increased 4-fold and 3-fold, respectively (*n* = 3, *p* < 0.05) (Figure 3B,C). 

### 2.4. Activation of the Wnt/β-Catenin Pathway in Static Culture Does Not Fully Mimic the Effect of Suspension Culture

**Figure 4:**
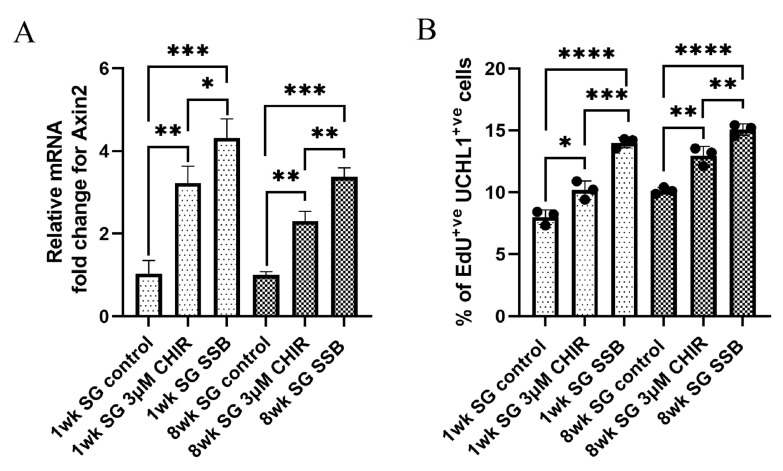
Activation of Wnt/β-catenin pathway with CHIR 99021 in static culture does not fully mimic the effects of stirred suspension bioreactor culture: (**A**) relative mRNA fold change of *Axin2* for 1- and 8-week-old spermatogonia (SG); (**B**) percentage of proliferating 1- and 8-week-old spermatogonia (SG). *n* = 3, mean ± SD. *p* > 0.05 (ns), * *p* ≤ 0.05, ** *p* ≤ 0.01, *** *p* ≤ 0.001, **** *p* ≤ 0.0001.

If Wnt/β-catenin is solely responsible for the enhanced proliferation seen in SSB culture, then activating the pathway in static culture would lead to similar levels of proliferation. To test that hypothesis, spermatogonia from 1- and 8-week-old pigs were cultured in SSBs at 120 rpm and in static cultures at 10% O_2_ tension with CHIR99021, a Glycogen Synthase Kinase 3β (GSK3β) inhibitor (Wnt activator) [50] for 14 days. Preliminary dose–response experiments showed that 3 µM CHIR99021 was the highest dose that could be used without negatively affecting cell viability. Upon treatment with 3 µM CHIR99021, transcription of *Axin2* was increased 3.2-fold for the treatment group and 4.3-fold for SSB, compared with control (*n* = 3, *p* < 0.05) for the 1-week-old spermatogonia (Figure 4A). For 8-week-old cells, *Axin2* transcription level increased 2.3-fold for treatment and 3.4-fold for SSB 120 rpm, compared with the control group (*n* = 3, *p* < 0.05) (Figure 4A). Proliferation levels were found to be higher in the treatment groups than in the control but were still lower than in SSB cultures (*n* = 3, *p* < 0.05) (Figure 4B). 

## 3. Discussion

Stirred suspension bioreactors have proven to be a robust culture system for the expansion of various stem cells [23,33,34,36]. Bioreactors can, in most cases, minimize or eliminate the shortcomings of conventionally employed static culture systems, allowing for easily scalable cell culture with minimal handling [12]. This is of particular importance when cells are grown for cell therapy applications [11]. With the advent of computational fluid dynamics modeling, it has become easier to fine-tune the shear environment allowing for more accurate scale-up strategies from smaller to larger bioreactors [51]. Despite these benefits, the application of a dynamic suspension culture system to spermatogonia remains relatively unexplored. In 2014, Zhang et al. reported the use of a dynamically simulated microgravity rotating cell culture system (RCCS) for culturing mouse spermatogonia for 14 days [52]. The cells were co-cultured with mitotically inactivated Sertoli cell feeders on fibrin scaffolds and grew as aggregates. However, this RCCS system did not allow opportunities for scalability and requires the use of feeder cells. We attempted to overcome these limitations with stirred suspension bioreactors. 

In stirred suspension bioreactors, cells grow as aggregates [23,25], and the aggregate size is determined by shear stress. Shear stress can be controlled by increasing or decreasing the rotation speed of the impeller, which correlates to the size of eddies [53]. The size of turbulent eddies is inversely proportional to rotation speed. Slower rotation speed leads to larger eddies, which are unable to break down larger aggregates. This allows the aggregates to grow too large and, as a result, fall out of suspension negating the beneficial effects of suspension culture. Too high of a rotation speed can generate eddies that are too small, leading to cell damage and death. At optimal rotation speed, the eddies are large enough to keep aggregates in suspension but small enough to impart shear stress to break down larger aggregates into smaller ones without damaging the cells [35]. According to our study, 120 rpm, which generated a maximum shear force of 3.9 dyne/cm^2^, was the optimal rotational speed to maintain spermatogonia in suspension and allow for proliferation. While the rotational speed of 80 rpm or 2.2 dyne/cm^2^ of maximum shear force was not detrimental to cell survival, it failed to elicit spermatogonia proliferation. In contrast, both 60 (1.5 dyne/cm^2^) and 140 rpm (4.8 dyne/cm^2^) were detrimental to cell survival, likely due to too low and too high of shear stress, respectively.

One key parameter in cell culture systems, which is often not reported but is critical for reproducibility, is the O_2_ tension that cells experience in the liquid phase. We measured O_2_ concentration in the liquid phase to be 5.2 ± 0.01 mM for static culture, which was lower than 6.3 ± 0.06 mM O_2_ concentration seen in bioreactor culture under atmospheric conditions. In 10% O_2_ cultures, O_2_ concentration in the bioreactor was around 1.3 ± 0.003 mM and in static culture was 1.1 ± 0.07 mM. Calgary is located at approximately 1084 m above sea level, and therefore, the O_2_ concentration in the gas phase of a tissue culture incubator operated at 37 °C under atmospheric conditions is 6.34 mM, and under 10% O_2_ conditions, it is 3.45 mM [14]. The solubility of O_2_, which is proportional to partial pressure, is rather low in a liquid phase (culture media). In addition, diffusion of O_2_ through the media, starting from the gas–liquid interphase to the bottom of the plate (where cells are in adherent culture) for non-stirred cultures is also considerably slow. The partial pressure of O_2_ in the ambient and 10% O_2_ incubators are, respectively, 16.34 kPa and 8.89 kPa; therefore, reduced O_2_ concentration in the static conditions, compared with the gas phase, is not surprising [14]. What is noteworthy here is that the O_2_ concentration in the liquid phase of bioreactors is higher than their static counterparts. Since the media in bioreactors is continuously stirred, the distribution of O_2_ throughout the liquid phase is accelerated, and as a result, the oxygen tension in bioreactors tends to be higher than in static culture [38]. 

As there is no established static culture system for pig spermatogonia, we compared mouse spermatogonia culture in bioreactors with the mouse static culture system. To the best of our knowledge, an established static culture system is only available for mouse spermatogonia. However, different from our observations with porcine spermatogonia, the stirred suspension bioreactor culture did not support the survival or proliferation of cultured mouse spermatogonia. One factor that may account for this difference could be that the mouse spermatogonia that we cultured in the bioreactors were not primary cells. To obtain sufficient cell numbers to load SSBs, these cells were expanded initially in static culture on mitotically inactivated STO feeder cells [6]. As a result, they may have become dependent on the presence of a feeder layer rendering them unsuitable for suspension culture. It may be necessary to transition primary mouse testicular cells directly onto a feeder-free suspension system without an intermediate static culture. Another way to address this issue would be to incorporate microcarriers, inert plastic beads that can be treated with different substrates such as gelatin or laminin to promote cell adhesion. Cells that do not actively form aggregates with each other may be adhered initially to such microcarriers and then cultured in bioreactors [33]. If feeder cells are essential, they can also be adhered to microcarrier beads and then mitotically inactivated to be incorporated in spermatogonia cultures. 

Spermatogonial stem cells (SSCs), precursors of spermatogonia, originate from bipotent primordial germ cells, which depend on oxidative phosphorylation (OXPHOS) [54,55,56,57], while adult cells primarily rely on high glycolytic flux and low mitochondrial activity for their metabolic needs [18,58,59,60]. We have shown that the transition to a more mature phenotype is initiated at 8 weeks of age, while 1-week-old pig spermatogonia mainly rely on OXPHOS fuelled by pyruvate consumption [32]. Glycolytic metabolism is required to secure carbon atoms for nucleotide, amino acid, and lipid biosynthesis, all required for rapid cell proliferation. At the same time, differences in metabolic flux cause a change in metabolite abundance within the cell, functioning as co-factors to alternate the epigenome and therefore transcriptional profile and ultimately cell function [61].

Lower oxygen tensions generally cause a relative shift in the contribution of glycolytic flux to the energy production of the cell. In SSCs, glycolysis has been shown to contribute to the enhancement of self-renewing circuits in vivo and in vitro via an increase in AKT activity [18,58]. AKT phosphorylation is suggested to be downstream of GFRα1 activation. Therefore, initiating a shift toward anaerobic metabolism via the decrease in oxygen partial pressure increases SSC maintenance circuits via AKT, similarly to what has been described in mouse models. In mice, an increase in AKT pathway activity has been shown to increase glycolytic flux and expression of GFRa1 [58]. PLZF and DAZL have a much broader expression pattern, and their regulation is most likely mainly regulated via post-transcriptional regulation, which can explain the fact that no change was observed on a transcriptional level. Aside from the physical benefits, bioreactors have also been shown to upregulate and maintain pluripotency markers while downregulating the differentiation potential of pluripotent stem cells [23,24,62] This is mediated by the bioreactors’ ability in modulating the Wnt/β-catenin pathway [25,34]. Previous studies have shown that the Wnt/β-catenin pathway is involved in the survival and proliferation of spermatogonia and is required for spermatogenesis [30,63,64]. Chassot et al. reported that constitutive activation of the Wnt pathway led to the proliferation of gonocytes [64]. Deletion of β-catenin in adult mouse testes caused a reduction of PLZF^+ve^ undifferentiated spermatogonia [30]. Similar to pluripotent cells [25], porcine spermatogonia also responded to shear forces in bioreactors by upregulating the Wnt/β-catenin pathway, characterized by nuclear β-catenin translocation and elevated *Axin2* transcription level. Although activation of the pathway in static culture with a small molecule activator CHIR 99021 caused increased proliferation, compared with controls, the proliferation levels observed after treatment were lower than in bioreactor culture. It is possible that, under the conditions tested, pathway activation with 3 um CHIR 99021 was incomplete [65]. In SSBs, exposure to fluid shear forces releases β-catenin from adherens junctions and also increases the levels of phosphorylated glycogen synthase kinase 3β, thus inhibiting β-catenin degradation. This inactivation of GSK3β is likely missing in static culture, even with CHIR 99021 treatment, causing both reduced *Axin2* transcription and spermatogonia proliferation. In addition, the physical benefits—namely, higher nutrient and gas exchange within the spermatogonia aggregates due to continuous hydrodynamic modulation, may also play an important role in spermatogonia survival and expansion in suspension culture.

Human pre-pubertal spermatogonia culture is challenging due to the scarcity of pre-pubertal tissue. It is also not clearly understood when metabolic transitions occur in humans. However, we have shown that early pre-pubertal human spermatogonia (1 year of age) have similar mitochondrial ultrastructure as 1-week-old pig spermatogonia [61]; therefore, human spermatogenic metabolic development might be similar to the pre-pubertal development in the pig. As the metabolic requirements of human pre-pubertal spermatogonia are still ambiguous, any culture techniques and especially bioreactor culture are still in their infancy. To summarize, we report a novel culture system for undifferentiated, non-rodent spermatogonia using stirred suspension bioreactors. Stirred suspension bioreactors allowed a scalable expansion of spermatogonia by both enhancing oxygen and mass transfer, as well as by modulating the Wnt/ β-catenin pathway within a controlled environment. Therefore, bioreactors could be an invaluable tool for future clinical applications such as cell therapy for treating infertility.

## 4. Materials and Methods

### 4.1. Spermatogonia Isolation and Enrichment

Testes from 1- and 8-week-old piglets were obtained from Sunterra Farms Ltd (Acme, AB, Canada) and the University of Alberta, Edmonton, AB. All procedures were performed with approval and under the oversight of the Animal Care Committee of the University of Calgary. Spermatogonia were harvested using a two-step enzymatic digestion process as previously described [66,67]. Briefly, testes were decapsulated and minced into ~1–2 mm pieces. These tissue pieces were then digested using collagenase IV (Sigma, Oakville, ON, Canada) (2 mg/mL), 0.25% trypsin–EDTA (Sigma, Oakville, ON, Canada), and DNase I (Sigma, Oakville, ON, Canada) (7 mg/mL) to obtain the starting cell population. Differential plating was performed with the starting cell population to enrich for spermatogonia. This enriched spermatogonia population was assigned to different experimental groups. All experiments were repeated using a minimum of three independently prepared cell suspensions.

### 4.2. Spermatogonia Culture 

Enriched porcine spermatogonia from 1- and 8-week-old piglets were suspended in spermatogonia culture media: Advanced Minimum Essential medium (Thermo Fisher Scientific, Mississauga, ON, Canada) supplemented with glial cell-derived neurotrophic factor (40 ng/mL), GFRα1 (25 ng/mL), and epidermal growth factor (20 ng/mL) [32] and were seeded into 10 mL SSBs (Corning Style Spinner Flask; NDS Technologies Inc., Vineland, NJ, USA) [25,51] at a concentration of 500 × 10^3^ cells per mL (which translated to 5 × 10^6^ cells suspended in 10 mL media in each SSB) and into 6-well plates (which translated to 1 × 10^6^ cells in 2 mL media in each well, a total of 5 wells were prepared for each sample totaling in 5 × 10^6^ cells) as static control. The SSBs were placed onto magnetic stirrers inside a cell culture incubator (37 °C which is 2 °C lower than the average body temperature (39 °C) of a domestic pig), which was preset for ambient O_2_ tension. The 1-week-old spermatogonia were stirred at 60, 80, 100, 120, and 140 rpm, while 8-week-old spermatogonia were cultured only at 120 rpm. Additionally, 120 rpm SSB and control static culture were performed in a tri-gas incubator, which was preset for O_2_ tension of 10%. The cells in SSBs and static control plates were cultured for a total of 14 days, with media changes at every 48 h. The cells were exposed to EdU (5-ethynyl-2′-deoxyuridine) for the last 12 h of culture, to quantify the proportion actively synthesizing DNA during that period, and were harvested using 0.25% trypsin EDTA. Cell viability was assessed using trypan blue exclusion assay.

### 4.3. Oxygen Measurement

Oxygen measurements were performed by using Oxygen Sensor Spots SP-PSt3-NAU (PreSens, Regensburg, Germany). The sensor was calibrated according to the manufacturer’s instructions. Measurements were performed for 1-week-old porcine spermatogonia cultured in static and 120 rpm SSBs at both ambient and 10% O_2_ tensions [32] for 48 h. 

### 4.4. Mouse Spermatogonia Culture

Spermatogonia from C57Bl/6 mice were cultured on a mitotically inactivated feeder layer of STO (SIM mouse embryo-derived thioguanine and ouabain resistant) embryonic fibroblasts [68] and were harvested with 0.25% trypsin–EDTA. The cells were then placed onto a 100 mm tissue culture dish with mouse spermatogonia culture media and incubated in a humidified tissue culture incubator (37 °C, 5% CO_2_) to allow the feeder cells to attach. After 1 h, only the non-adhered spermatogonia were collected and seeded into a 10 mL SSB (5 × 10^6^ cells/ SSB). A conventional feeder-based culture was used as a static control. The mouse spermatogonia culture medium was composed of minimum essential medium α (Fisher Scientific, Ottawa, ON, Canada) supplemented with 0.2 % bovine serum albumin, transferrin (10 µg/mL), insulin (5 µg/mL), free fatty acid mixture (7.6 µ eq/mL), sodium selenite (3 × 10^−8^ M), 2-mercaptoethanol (50 µM), HEPES (10 mM), putrescine (60 µM), glutamine (2 mM), 1% penicillin/streptomycin, recombinant human GDNF (20 ng/mL), recombinant rat GFRA1 (75 ng/mL), and FGF2 (1 ng/mL) [39]. The SSBs were stirred at 120 rpm and cultured for 14 days as before.

### 4.5. CHIR Treatment

Porcine spermatogonia from 1- and 8-week-old piglets were seeded into a 6-well plate, and the media was supplemented with 3 μM CHIR 99021 (Bio Techne, Toronto, ON, Canada) dissolved in DMSO (0.03% DMSO in medium). The control group was treated with an equal amount of DMSO. The culture was carried out in a hypoxic incubator. The total duration of culture was 14 days with media changes every day. Each time media was supplemented with 3 μM CHIR 99021. The cells were then harvested and analyzed for proliferation and *Axin2* transcription level.

### 4.6. EdU Incorporation Assay

Cells were pulsed with 5 μM EdU (5-ethynyl-2′-deoxyuridine), a nucleoside analog incorporated into DNA during replication, for the last 12 h of all cultures. EdU uptake was visualized using Click-iT EdU Alexa Fluor 647 imaging kit as per manufacturers protocol (Thermo Fisher Scientific, Mississauga, ON, Canada). 

### 4.7. Immunofluorescence

Testis tissue was fixed in 4% paraformaldehyde (PFA) at 4 °C overnight, dehydrated with a gradient series of ethanol, and embedded in paraffin wax to prepare sections of 5 µm thickness. Cells were fixed with 2% PFA at room temperature for 15 min and stained for EdU according to instructions by the manufacturer. Both the cells and the tissue were then blocked with CAS-Block (Thermo Fisher Scientific, Mississauga, ON, Canada) and incubated overnight with anti-UCH-L1 (ubiquitin C-terminal hydrolase L1; 1:100 dilution; Abcam, Waltham, MA, USA), anti-vimentin (1:500, Sigma, Oakville, ON, Canada) or anti-β-catenin (1:200 dilution; Abcam, Waltham, MA, USA). The samples were then incubated with secondary antibodies conjugated with Alexa Fluor 488 and 555. The nuclei were stained with DAPI (4′,6-diamidino-2-phenylindole), and the samples were analyzed using fluorescence microscopy. The number and percentage of cells were quantified by counting approximately 350 cells per sample.

### 4.8. Quantitative Reverse Transcription Polymerase Chain Reaction (qRT-PCR)

The transcription levels of spermatogonia markers, upstream and downstream targets of Wnt/ β-catenin (Table 3), were assessed via qRT-PCR after fluorescent-activated cell sorting (FACS) sorting of enriched spermatogonia as described in [32]. RNA was extracted from 1 × 10^5^ cells using RNeasy Micro Kit (QIAGEN, Toronto, ON, Canada). Reverse transcription was performed using SuperScript™ IV VILO™ Master Mix (Thermo Fisher Scientific, Mississauga, ON, Canada). In addition, the 7.500 Fast Real-Time PCR System (Applied Biosystems, Waltham, MA, USA) was used, with SsoFast Eva Green Supermix with Low ROX (Bio-Rad laboratories, Mississauga, ON, Canada) for qPCR. Relative expression is calculated using 2^−ΔΔCT^. Statistical analysis was performed on the mean of ΔΔCt.

### 4.9. Maximum Shear Stress Calculation

Maximum shear stress in a 10 mL bioreactor was calculated using equations from Gareau et al. 2014 [34]. The required dimensions of 10 mL bioreactors are stated in Table 4.

### 4.10. Statistical Analysis

All data reported are from at least three independent experiments performed with separately prepared enriched spermatogonia populations from different animals (*n* = 3). The data were analyzed by unpaired two-tailed *t*-tests to compare two groups and one-way ANOVA for more than two groups with the Tukey multiple comparisons test. A value of *p* < 0.05 was set as the limit of statistical significance. Statistical analyses were performed using the GraphPad Prism 8 software (San Diego, CA, USA).

## Figures and Tables

**Table 1:** Calculation of O_2_ tension and concentration in gas phase at ~1084 m elevation (see Supplementary Methods).

Conditions	O_2_ Tension Pre-Set for the Incubator	Partial Pressure of O_2_	True O_2_ Tension (at 1084 m Altitude)	Concentration in Gas Phase
Ambient	20.9%	16.34 kPa	18.4%	6.34 mM
10% O_2_	10%	8.89 kPa	10%	3.45 mM

**Table 2:** O_2_ measurements in media via spot sensors (Supplementary Methods).

Conditions	O_2_ Tension	Concentration
Static ambient O_2_	14.9 ± 0.04%	5.2 ± 0.01 mM
SSB ambient O_2_	18.2 ± 0.17%	6.3 ± 0.06 mM
Static 10% O_2_	3.28 ± 0.22%	1.1 ± 0.07 mM
SSB 10% O_2_	3.74 ± 0.01%	1.3 ± 0.003 mM

**Table 3:** Primer sequences used for qPCR.

Gene Name	Direction	Sequence
*GFRα1*	Forward	CATCTGCAGATCTCGCCTGGC
	Reverse	GCCAAAGGCTTGAATTGCATTTTTGAGAC
*PLZF*	Forward	GGG TGC ATA CAG GTG AGA AGC C
	Reverse	CAC ACA TAG CAC AGG TAG AGG TAC GTC
*E-cadherin*	Forward	GAGAAGAGGACCAGGACTTTGACTTGAG
	Reverse	TCACTATCAGCTGCCTTCAGGTTTTCATC
*Axin2*	Forward	GAGGGAGAAATGCGTGGATA
	Reverse	GGTTTCAGCTGCTTGGAGAC
*DAZL*	Forward	GTT ATT CCT CCG GCT TAT ACA GCT G
	Reverse	GAT ACC ACT GTC TGT ATG CTT CGG TC
*HPRT1*	Forward	GAAGAGCTACTGTAATGACCAGTCAACGG
	Reverse	TCATTGTAGTCAAGGGCATAGCCTACC

All sequences are listed in the 5′ to 3′ direction.

**Table 4:** Dimensions of 10 mL stirred suspension bioreactor.

Parameters	Dimensions
Media volume	0.00001 m^3^
Impeller width	0.0054 m
Impeller diameter	0.0254 m
Bioreactor diameter	0.032 m

## Data Availability

The data that support the findings of this study are available on request from the corresponding author.

## Supplementary Materials

The following are available online at https://www.mdpi.com/article/10.3390/ijms222413549/s1.
