# Supplementary material for: The Proliferation of Pre-Pubertal Porcine Spermatogonia in Stirred Suspension Bioreactors Is Partially Mediated by the Wnt/β-Catenin Pathway
Source: Int J Mol Sci. 2021 Dec 17;22(24):13549. doi: 10.3390/ijms222413549 (PMC8708394; doi:10.3390/ijms222413549)
Supplement: Supplementary file 1 [file ijms-22-13549-s001.zip › Sakib et al. supplementary figures-edited.pdf]

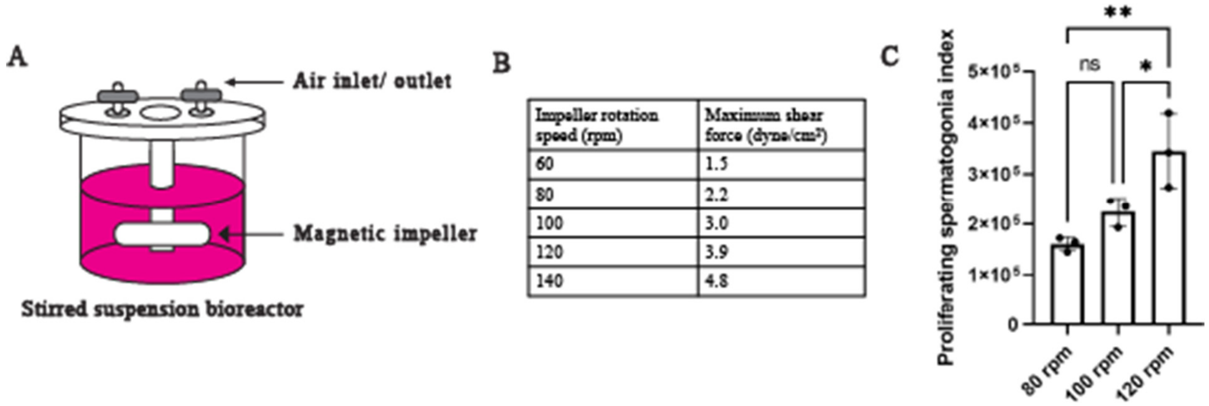

Impeller rotation speed (rpm)	Maximum shear force (dyne/cm²)
60	1.5
80	2.2
100	3.0
120	3.9
140	4.8

Figure S1. Stirred suspension bioreactor culture of porcine spermatogonia: (**A**) schematic of a 10 mL stirred suspension bioreactor; (**B**) maximum shear forces generated in the stirred suspension bioreactors; (**C**) proliferating spermatogonia index (number of EdU^{+ve} UCHL1^{+ve} cells) for 1-week-old porcinespermatogonia. n = 3, mean ± SD. $p > 0.05$ (ns), *$p \leq 0.05$, **$p \leq 0.01$.

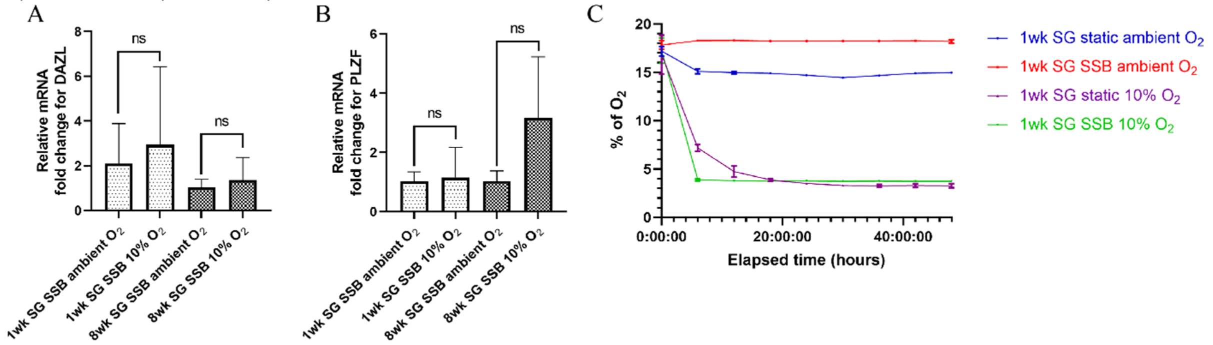

Figure S2. Gene expression of PLZF and DAZL: (**A**) relative mRNA fold change of DAZL for 1- and 8-week- old spermatogonia (SG); (**B**) relative mRNA fold change of PLZF for 1- and 8-week-old spermatogonia (SG). n = 3, mean ± SD. $p > 0.05$ (ns), *$p \leq 0.05$, **$p \leq 0.01$, ***$p \leq 0.001$, ****$p \leq 0.0001$. (**C**) Percentage of O_2 measured via Oxygen Sensor Spots over the course of 48 h.

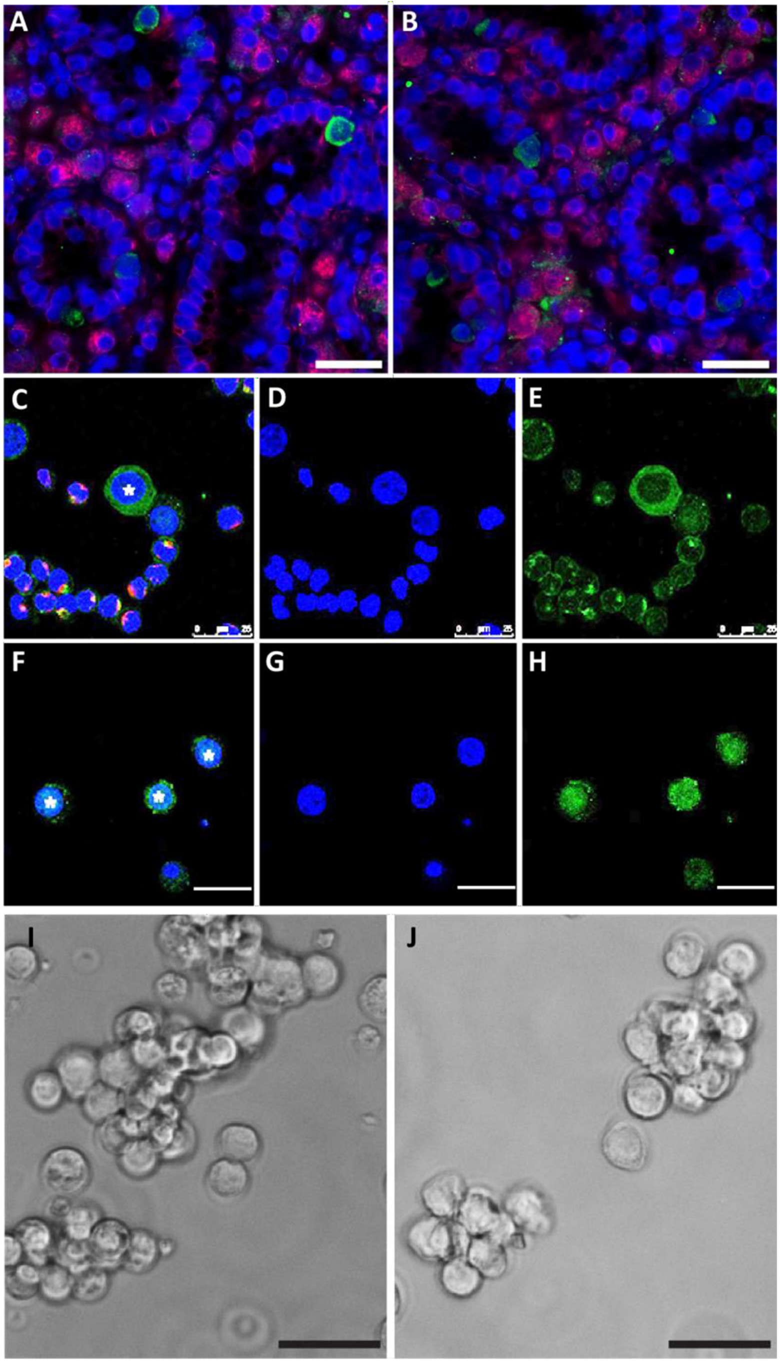

Figure S3. Immunofluorescence image of testis tissue and cells labeled for vimentin, UCHL1, and β-catenin, as well as brightfield images of spermatogonia clusters: (**A**,**B**) immunofluorescence image of UCHL1 (green) and vimentin (red) in 1-week-old (**A**) and 8-week-old testes (**B**); (**C**–**H**) immunofluorescence image of β-catenin(green) and vimentin (red): (**C**–**E**) static culture showing cytoplasmic β-catenin and (**F**–**H**) bioreactor culture showing nuclear β-catenin; (**I**,**J**) Brightfield image of 1-week-old (**I**) and 8-week-old (**J**) spermatogonia clusters after bioreactor culture. Scale bar 25 μm.
