# Supplementary material for: The Proliferation of Pre-Pubertal Porcine Spermatogonia in Stirred Suspension Bioreactors Is Partially Mediated by the Wnt/β-Catenin Pathway
Source: Int J Mol Sci. 2021 Dec 17;22(24):13549. doi: 10.3390/ijms222413549 (PMC8708394; doi:10.3390/ijms222413549)
Supplement: Supplementary file 1 [file ijms-22-13549-s001.zip › Sakib et al. Supplementary methods-edited.pdf]

Supplementary Methods: Measurement of O_2 concentration in liquid and gas phases

Calculation of oxygen tension at 1084 m of altitude:

The atmospheric pressure in Calgary, AB, Canada, located at 1084 m above sea level, is approximately 88.9 kPa [37]. According to Dalton's law, the partial pressure values of all relevant gases are equal to the atmospheric total pressure [14, 38]. In an appropriately humidified tissue culture incubator at atmospheric O_2 tension of 20.9%, 5% CO_2, and 37 °C, the partial pressure of water pH_2O is 6.28 kPa (constant) [14], and the partial pressure of pCO_2 is 4.44 kPa (5% of 88.9 kPa). The remaining dry room airpressure is 88.9-6.28-4.44= 78.18 kPa. Thus, the partial pressure of oxygen pO_2 is 16.34 kPa (20.9% of the remaining dry air pressure 78.18 kPa). This translates to 18.4% of atmospheric pressure (88.9 kPa). In a tri-gas incubator pre-set for 10% O_2 tension, the system measures % of O_2 in real time and keeps it constant at 10%. The partial pressure of 10% O_2 is then 8.89 kPa (10% of atmospheric pressure).

Estimation of oxygen concentration in the gas phase:

The ideal gas law can be used to estimate the concentration of gas as follows:

$$PV=nRT \tag{1}$$

where P= pressure of the gas (kPa); V= volume of the gas (L); n= number of molecules of gas (mol);R= ideal gas content (8.31 J K^{-1} mol^{-1});T= temperature (K).

Using Equation (i), the concentration of O_2 in the gaseous phase of in the incubator set for ambient culture (pO_2=16.34 kPa) is 6.34 mM, and the concentration of O_2 for the hypoxic culture (pO_2=8.89 kPa) is 3.45 mM.

Estimation of oxygen concentration in media (liquid phase):

The O_2 percentages (% v/v) from the spot sensors were used to determine the volume of O_2 in 1 L of media. Using Equation (i), the molar volume (Vm=V/n=RT/P=28.99 L) at 37°C and a pressure of 88.9 kPa were determined.The number of moles from the volume of O_2 was then determined using the molar volume, which was then used to determine molarity.
